# Unveiling the Spectrum of Liver Endurance in Systemic Lupus Erythematosus: A Single-Center Experience From Kashmir, India

**DOI:** 10.7759/cureus.96374

**Published:** 2025-11-08

**Authors:** Zeeshan Wani, Shaheen Nazir, Afrah Nasir, Aasif Iqbal, Chhagan Bihari

**Affiliations:** 1 Gastroenterology, Super Speciality Hospital, Srinagar, IND; 2 Gastroenterology and Hepatology, Sher-i-Kashmir Institute of Medical Sciences, Srinagar, IND; 3 Pathology, Institute of Liver and Biliary Sciences, New Delhi, IND

**Keywords:** acute liver failure (alf), budd chiari syndrome (bsc), drug induced liver injury (dili), liver involvement, lupus hepatitis, nodular regenerative hyperplasia (nrh), nonalcoholic fatty liver disease (nafld), portal vein thrombosis (pvt), systemic lupus erythematosus (sle)

## Abstract

Background

Systemic lupus erythematosus (SLE) presents myriad challenges to gastroenterologists, although clinically significant liver involvement is uncommon. Literature regarding liver involvement remains variable, and the scope to illuminate this issue is vast, necessitating further study.

Aims and objectives

The purpose of this study was to assess and categorize the liver involvement in SLE and to evaluate treatment response.

Methods

Our study was a retrospective observational study. Diagnosed cases of SLE referred to the Department of Gastroenterology were evaluated for liver involvement. Detailed history, clinical examination, and biochemical profile were recorded. Imaging, liver biopsy, and/or endoscopic gastroduodenoscopy were used as adjuncts wherever needed. After proper evaluation, patients were categorized according to liver involvement, and treatment response was assessed.

Results

Out of 96 patients with SLE referred to gastroenterology, 32 had liver abnormalities. Six (12.5%) patients had non-alcoholic fatty liver disease, four (12.5%) had nodular regenerative hyperplasia, two (6.25%) had lupus hepatitis, five (15.62%) had drug-induced liver injury, and one case each (3.12%) had hepatic infarction, hepatitis B, and hepatitis C. Autoimmune hepatitis (AIH) was seen in five (15.62%) patients, primary biliary cholangitis (PBC) was seen in one (3.12%), and AIH-PBC overlap was seen in three (9.375%) patients. Treatment was tailored according to the diagnosis, and response was assessed. One patient with hepatic infarction presenting as ALF died.

Conclusion

Liver perturbations may arise from basic diseases or side effects during treatment or unrelated factors. It is a multifaceted interplay between autoimmune mechanisms, vascular complications, and drug-related hepatotoxicity. Recognizing diverse presentations and understanding diagnostic intricacies are crucial for clinicians managing SLE patients with hepatic involvement, enabling better treatment outcomes.

## Introduction

Systemic lupus erythematosus (SLE) is a complex and multifaceted autoimmune disorder that can affect multiple organs, including the liver [1,2]. The liver's involvement in SLE, characterized by elevated liver enzymes, hepatomegaly, and jaundice, can mimic other liver diseases, making accurate diagnosis and timely intervention crucial [3,4]. Establishing the cause of liver involvement in SLE manifesting as abnormal liver enzymes poses a challenge to clinicians, even though often these aberrations are clinically trivial and transient [3,4]. It is critical to subcategorize and evaluate them properly, as most of them have effective treatment.

Liver involvement in SLE could be due to lupus hepatitis, a distinct entity due to the basic disease, which manifests as elevated liver enzymes, hepatomegaly, and jaundice. Discerning lupus hepatitis from other liver diseases remains pivotal for accurate diagnosis and timely intervention [5]. Beyond lupus hepatitis, SLE may coexist with autoimmune hepatitis (AIH), primary biliary cholangitis (PBC), or overlap, further complicating the diagnostic panorama [6]. The treatment landscape for SLE often involves immunosuppressive regimens, which can give rise to unique challenges in management. Hepatic complications, such as drug-induced liver injury (DILI) and immunosuppression-related infections, necessitate vigilant monitoring and a nuanced management approach [3,4,7]. With the epidemic of metabolic syndrome worldwide, with non-alcoholic steatohepatitis (NASH) being now highly prevalent, many SLE patients inevitably develop a NASH overlay on their underlying disease [3]. Viral hepatitis B or C could be another reason for liver involvement, as has been seen worldwide, and needs to be excluded [4]. Vascular causes of liver abnormalities, such as Budd-Chiari syndrome and portal vein thrombosis (PVT), were observed only rarely [8]. Recognition of all these concomitant diseases is essential to guide tailored therapeutic strategies. Overall, data on liver biochemical and clinical abnormalities among SLE patients worldwide are not profound and especially scarce in our subcontinent. In this context, the present study aimed to investigate, categorize, and classify liver involvement in SLE based on its underlying etiologies, with the ultimate goal of establishing a systematic classification framework to ensure comprehensive diagnosis and treatment. Additionally, we sought to evaluate the treatment outcomes of these patients in our regional setting.

## Materials and methods

Study design and setting

This retrospective observational study was conducted at the Department of Gastroenterology, Super Speciality Hospital, Sheerin Bagh (SSH), a tertiary care institute in Kashmir, India.

Study period and population

The study included diagnosed SLE patients referred to the Department of Gastroenterology from December 2021 to December 2022. Diagnosis was based on the SLICC/ACR 2012 classification criteria [1] and confirmed by the treating rheumatologist.

Inclusion criteria

The study included adult patients with biochemical or imaging evidence of liver involvement, which was defined as the presence of transaminitis and/or a dysmorphic liver on imaging, as well as features of portal hypertension such as esophageal varices or ascites.

Exclusion criteria

The study excluded individuals who were under 18 years old, had prior liver disease of other etiologies, had known malignancies or hepatocellular carcinoma, were active IV drug or alcohol abusers, had severe systemic illness or sepsis, or were pregnant or lactating females.

Sampling technique

Consecutive SLE patients attending the Department of Gastroenterology at SSH were enrolled in the study.

Diagnostic approach

The diagnostic approach involved a series of steps. Firstly, patients with elevated liver enzymes underwent hepatitis B and C serology testing. Secondly, autoimmune markers were evaluated, and, if positive, liver biopsies were performed to document disease. If autoimmune markers were negative, vascular involvement was evaluated. If an inciting drug was identified, it was discontinued, and the patient was monitored for enzyme improvement. If enzyme improvement was observed, a diagnosis of DILI was made. A liver biopsy was performed in all probable cases of AIH, PBC, or overlap syndromes, in those in whom there was no improvement after withdrawing suspected causative drugs, and in those in whom the diagnosis remained uncertain after preliminary workup.

Study procedure

The study procedure involved recording demographic characteristics, taking a detailed history and physical examination, and conducting baseline investigations such as liver function tests (LFT), kidney function tests (KFT), and complete blood counts (CBC). Additionally, Doppler ultrasound of the abdomen, etiologic profile, contrast-enhanced computed tomography (CECT) of the abdomen, endoscopic gastroduodenoscopy, and liver biopsy were performed where needed.

Fatty liver grading

Fatty liver was graded based on the degree of hepatic echogenicity on ultrasound [9]. Grade 1 was characterized by increased hepatic echogenicity, grade 2 by obscuring periportal echogenicity, and grade 3 by obscuring diaphragmatic echogenicity. It was correlated with controlled attenuation parameter in all NASH patients and with biopsy where needed.

Patient categorization

Patients were categorized into three groups based on the etiology of liver involvement. The first group consisted of patients with SLE-related liver involvement, which included lupus hepatitis, AIH, and PBC. The second group consisted of patients with SLE-unrelated liver involvement, which included viral hepatitis and DILI. The third group consisted of patients with conditions that had a possible relation to SLE, such as non-alcoholic fatty liver disease and vascular thrombosis.

Statistical analysis

SPSS version 20 (IBM Corp., Armonk, NY) was used for analysis. Data were analyzed using descriptive statistics, as the study primarily focused on observational findings. Continuous variables were expressed as mean (SD) and range. Categorical variables were expressed as frequencies and percentages.

## Results

A total of 96 SLE patients were assessed, but 64 were excluded due to unrelated gastrointestinal symptoms. The final study cohort consisted of 32 patients (all females) with SLE and liver involvement. Mean age was 36.68 ± 10.80 years, with 53.4% of patients between 30 and 50 years of age. Demographic features and baseline parameters are summarized in Table 1.

**Table 1:** Demographic features and baseline parameters BIL, bilirubin; AST, aspartate aminotransferase; ALT, alanine aminotransferase; ALP, alkaline phosphatase; TP, total protein; ALB, albumin; Hb, hemoglobulin; TLC, total leucocyte count; PLT, platelet; Sr IgG, serum immunoglobulin

Parameters	Reference range	Minimum	Maximum	Mean	SD
Age (years)	>18	20	60	36.68	10.80
BIL (mg/dL)	0.1 to 1.2	0.50	18.00	3.3550	4.37648
AST (U/L)	8-35	12	8,083	503.53	1434.761
ALT (U/L)	8-35	32	9,562	606.66	1704.948
ALP (IU/L)	44-147	68	652	247.72	150.433
TP (g/dL)	6-8	4.52	8.00	6.8725	0.86117
ALB (g/dL)	3.4-5.4	1.20	4.60	3.0534	0.73184
HB (g/dL)	12-15.5	6.6	14.0	9.772	1.8476
TLC (cells/micL)	4,000-11,000	2.8	7.1	4.916	1.1767
PLT (cell/micL)	150,000-450,000	3.4	346.0	119.844	74.1432
Sr IgG (mg/dL)	600-1,600	1,400	4,385	2293.91	1086.240
Fibro-scan (Kpa)	2-7	4.3	24.0	11.476	5.1363

Symptoms included abdominal discomfort (73%), jaundice (53%), and fever (40%). Liver enzymes were elevated in all patients (median ALT: 90 IU/L, AST: 60 IU/L). Complications included cirrhosis (25%), esophageal varices (22%), and vascular involvement with high enzyme levels (12.5%).

Liver involvement patterns

Liver involvement in SLE patients exhibited diverse patterns. Parenchymal disease was most common (87.5%), while vascular disease (12.5%) followed a more aggressive course. AIH/overlap syndrome and NASH were also the most common. No significant differences were found between the groups. One of our patients was excluded as she turned out to have ascites due to Tjalma syndrome. The patterns of liver involvement are summarized in Table 2, and laboratory and imaging findings are given in Table 3. We observed a varied pattern of enzyme elevation among patients with different liver conditions. Mild elevations in liver enzymes were observed among patients with viral hepatitis, AIH, NASH, PVT, and in one case of lupus hepatitis. In contrast, moderate to severe elevation was noted in one patient with lupus hepatitis and another with hepatic infarction, which showed the highest enzyme levels (8,000-9,000 range). Six patients presented with cirrhosis: two with nodular regenerative hyperplasia (NRH), two with NASH, one with chronic hepatitis B, and one with AIH. Hepatomegaly was observed in 40% (13/32) of patients, with it being the most common finding. Splenomegaly was noted in 28.12% (9/32) of patients, and ascites was present in three patients.

**Table 2:** Patterns of liver involvement AIH, autoimmune hepatitis; ALF, acute liver failure; BCS, Budd-Chiari syndrome; DILI, drug-induced liver injury; EHPVO, extrahepatic portal vein obstruction; HBV, hepatitis B virus infection; HCV, hepatitis C virus infection; NASH, nonalcoholic steatohepatitis; NRH, nodular regenerative hyperplasia; PBC, primary biliary cholangitis; PVT, portal vein thrombosis; SLE, systemic lupus erythematosus

		Diagnosis	N=32	N %
Parenchymal involvement	SLE-related	AIH	5	15.62%
		AIH-PBC	3	9.37%
		PBC	1	3.12%
		Lupus Hepatitis	2	6.25%
		NRH	4	12.5%
	SLE-unrelated	HBV	1	3.12%
		HBV	1	3.12%
		DILI	5	15.62%
	SLE-potentially related	NASH	6	18.75%
Vascular involvement		EHPVO	1	3.12%
		PVT	1	3.12%
		BCS	1	3.12%
		Hepatic infarct as ALF	1	3.12%

**Table 3:** Laboratory and imaging findings EHPVO, extrahepatic portal vein obstruction; HBV, hepatitis B virus infection; HCV, hepatitis C virus infection; HVT, hepatic vein thrombosis; MRCP, magnetic resonance cholangiopancreatography; PVT, portal vein thrombosis

Parameter	Status	N (%)
Anti-nuclear antibody (ANA)	Positive	32 (100)
Anti-double-stranded DNA	Positive	32 (100)
Anti ribonucleoprotein (ARNP)	Negative	29 (90.6)
	Positive	3 (9.4)
C3/C4	Normal	19 (59.4)
	Decreased	13 (40.6)
Hepatitis serology (HBV and HCV)	Negative	30 (93.8)
	HBV + (HBV DNA-)	1 (3.1)
	HCV + (HCV RNA-)	1 (3.1)
Anti-mitochondrial antibody (Anti-AMA)	Negative	28 (87.5)
	Positive	4 (12.5)
Anti-smooth muscle antibody (ASMA)	Negative	27 (84.4)
	Positive	3 (9.4)
	Not available	2 (6.2)
Anti-liver kidney microsomal antibody (anti-LKM)	Negative	27 (84.4)
	Positive	3 (9.4)
	Not available	2 (6.2)
MRCP	Normal	30 (100)
	Not available	2 (100)
CECT of the abdomen with CT angiography	HVT/PVT/EHPVO	3 (9.4)
	Normal	19 (59.3)
	Not available	10 (31.25)

Histopathological findings

**Figure 1:**
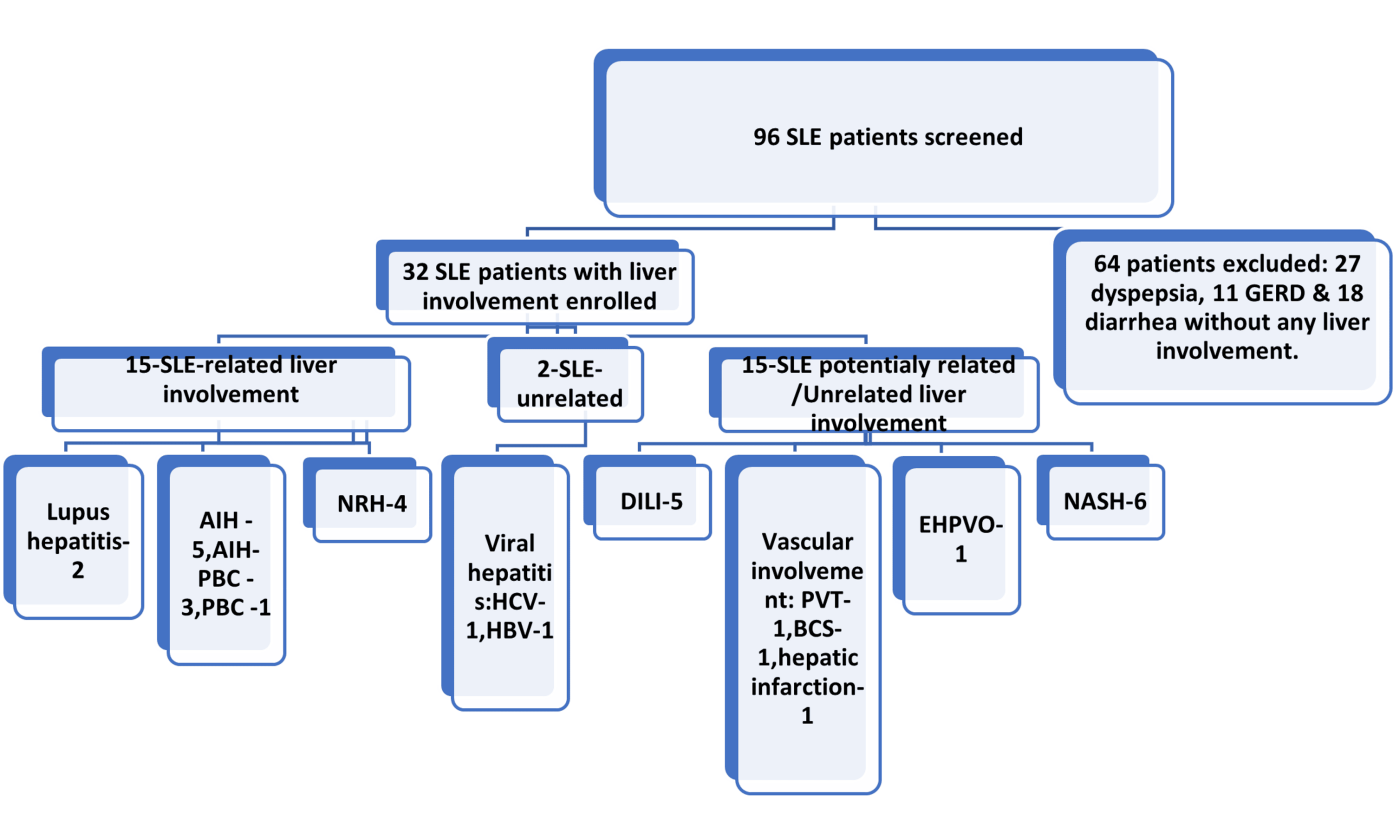
A concept diagram AIH, autoimmune hepatitis; ALF, acute liver failure; BCS, Budd-Chiari syndrome; DILI, drug-induced liver injury; EHPVO, extrahepatic portal vein obstruction; GERD, gastroesophageal reflux; HBV, hepatitis B virus infection; HCV, hepatitis C virus infection; NAFLD, nonalcoholic fatty liver disease; NASH, on-alcoholic steatohepatitis; NRH, nodular regenerative hyperplasia; PBC, primary biliary cholangitis; PVT, portal vein thrombosis; SLE, systemic lupus erythematosus *Numbers are number of patients

**Figure 2:**
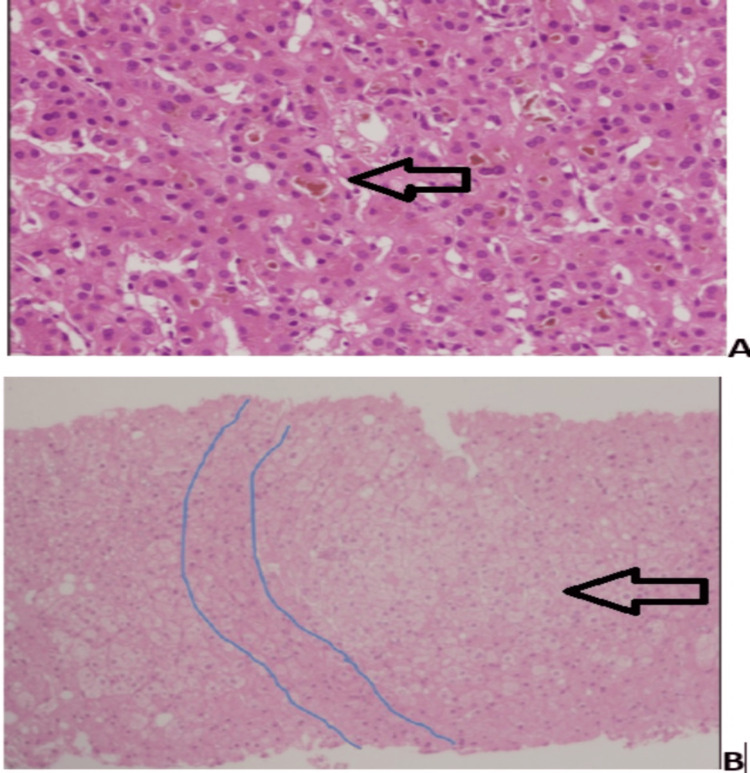
Histopathological findings of NRH (A) H&E-stained section, x200, showing bland cholestasis (arrow). (B) H&E-stained section, x100, showing regenerative group of hepatocytes, encircled by compressed hepatocytes (depicted), consistent with NRH (arrow). NRH, nodular regenerative hyperplasia

Liver biopsy was performed in 19 patients, revealing a range of histopathological findings (Table 4). The most common findings were lymphocytic infiltration (n = 6, 60%), interface hepatitis (n = 5, 50%), and lobular inflammation (n = 4, 40%). Fibrosis was present in seven (70%) patients, with two (20%) patients showing cirrhosis. Figure 1 shows a concept diagram of the whole results, and Figure 2 illustrates the histopathological features of select cases, showcasing representative examples of liver involvement.

**Table 4:** Liver biopsy findings in all cases AIH, autoimmune hepatitis; DILI, drug-induced liver injury; NASH, nonalcoholic steatohepatitis; NRH, nodular regenerative hyperplasia; PBC, primary biliary cholangitis

Liver Biopsy	N=32	%
Biopsy not done	11	24.37%
AIH	5	15.62%
PBC	1	3.125%
AIH-PBC overlap	3	9.37%
NASH	4	12.5%
NRH	4	12.5%
DILI	2	6.25%
Lupus Hepatitis	2	6.25%

Clinical presentation

The most common presentation was transaminitis (71.87%). Other notable findings included chronic liver disease (CLD) features on imaging in six (18.75%) patients and acute abdominal complications in three patients, such as hepatic infarction with acute liver failure, Budd-Chiari syndrome, and PVT with mesenteric extension, leading to gut gangrene that required surgical resection.

Treatment outcomes

Treatment outcomes for liver conditions varied based on underlying etiology. Hepatitis C patients recovered with antiviral therapy, while hepatitis B patients with established cirrhosis remained stable on tenofovir alafenamide and beta blockers. Patients with Budd-Chiari syndrome and PVT showed significant improvement with anticoagulation after ICU management. Lupus hepatitis patients responded well to pulse steroid therapy. AIH, overlap syndrome, and PBC patients were treated with steroids, immunosuppressive therapy, and ursodeoxycholic acid according to standard guidelines. Among 15 patients, treatment outcomes were favorable in most cases. All patients received corticosteroids, with 10 (66.7%) also receiving immunosuppressive agents, primarily azathioprine (40%). Liver function tests improved in 11 (73%) patients, with two (13%) patients experiencing complete normalization of liver enzymes. One patient with hepatic infarction and acute liver failure died after five days of ICU management. Our findings highlight the importance of early recognition and treatment of liver involvement in SLE patients to prevent long-term liver damage and improve outcomes.

## Discussion

SLE is a multi-systemic autoimmune disorder characterized by a wide gamut of clinical presentations [3]. Although clinically significant liver involvement is uncommon, subclinical liver involvement is not rare [3,4]. Hepatic involvement is a daunting task for clinicians owing to the paucity of structured supporting literature. Most studies evaluating liver involvement in SLE, including our own, are limited by small sample sizes and retrospective designs, which preclude robust estimation of true prevalence rates. As a result, direct prevalence comparisons across studies or populations are methodologically unreliable. Nevertheless, we have attempted to present comparative proportions from selected cohorts to provide a contextual understanding of the relative frequency of various hepatic manifestations. These comparisons should be interpreted with caution, as heterogeneity in study design, population characteristics, diagnostic criteria, and reporting practices may significantly influence reported rates. While such percentages offer a tentative framework for discussion, they are not a substitute for high-quality epidemiological data.

Although males with SLE have been reported to have more liver involvement [7], our study, comprising only female patients, did not allow for comparison. We found no distinguishing factors, such as age, BMI, or symptoms, between liver involvement subcategories, consistent with previous studies [5-7]. The mean age at presentation was 36.68 ± 10.80 years, consistent with previous studies [7]. Asymptomatic transaminitis is one of the most common liver manifestations [3,4,7].

SLE-related liver involvement

In our cohort of 32 patients, only 2/32 (6.25%) were diagnosed with lupus-related liver disease after excluding other potential etiologies. This rate is consistent with other cohort studies, which report a prevalence of lupus hepatitis ranging from 3% to 25% [10-12], indicating that our findings fall within the reported range, though on the lower end. These patients, aged 20 with newly diagnosed SLE and a 42-year-old with long-standing disease, exemplified the diverse presentation of lupus-related liver disease, presenting with significantly elevated (>10-fold) and mild liver enzyme elevations, respectively, as reported in previous literature [7,13]. The latter patient posed a diagnostic challenge, failing to improve after azathioprine withdrawal, and ultimately required a liver biopsy. Liver biopsy findings were consistent with lupus hepatitis, aligning with previously documented histopathological features [10]. Both patients received pulse intravenous steroids, leading to improved symptoms and resolved transaminitis. Notably, anti-ribosomal P antibodies were negative despite their association with lupus hepatitis [11,12]. Immunologic mechanisms drive the inflammatory process, and differentiating lupus hepatitis from other liver diseases, such as viral hepatitis, is vital for tailored management. Serological markers, such as ANA and anti-double-stranded DNA, support diagnosis, and timely immunosuppressive therapy is crucial [10].

Around 15% of SLE patients may experience overlapping AIH, adding complexity to hepatic manifestations [3,4,7]. Distinguishing between SLE-related liver disease and primary AIH can be challenging, requiring meticulous evaluation of serological markers, histopathology, and clinical features. In our study, 15.62% (5/32) of patients had AIH, 9.37% (3/32) had AIH/PBC overlap, and 3.12% (1/32) had PBC. This rate is comparable to those reported in cohort studies, which suggest AIH-SLE overlap rates between 10-20% [4,6,7], supporting the generalizability of our findings. All patients were diagnosed after consistent positive markers and biopsies. Among AIH patients, one had features of CLD and high fibrosis, while another had concomitant DILI. Our patient with PBC was diagnosed with both conditions almost simultaneously, differing from previous studies, which have mostly reported PBC preceding SLE in up to 75% of cases [6]. This variance may reflect population differences or timing of SLE diagnosis.

We identified NRH in 4/32 (12.5%) patients. Vaiphei et al. reported NRH in 43% in a small sample of SLE patients with enzyme abnormalities, suggesting that our percentages are lower than that in some select groups but still high relative to the general population [14,15], further suggesting either regional difference or referral bias in our center. NRH a rare hepatic manifestation characterized by transformation of hepatic parenchyma into small regenerative nodules [10]. NRH prognosis depends on the underlying disease and portal hypertension severity. In SLE, NRH may be attributed to portal vasculopathy or immunosuppressive therapy effects [14,15]. Most of our patients were on azathioprine and prednisone and presented with anemia, ascites, and splenomegaly. Imaging and liver biopsy revealed characteristic NRH features, including mild, nonspecific regenerative changes without fibrosis or cirrhosis [10,14,16]. This highlights the importance of considering NRH in the differential diagnosis of hepatic involvement in SLE patients, particularly those on immunosuppressive therapy.

Liver involvement potentially related or unrelated to SLE

Fatty liver in SLE patients can be due to disease-related or unrelated causes, including drug-induced toxicity from methotrexate, NSAIDs, and high-dose steroids [16,17]. Some studies suggest that SLE itself can cause fatty liver changes, reversible with steroid use [12,18]. In our study, 6/32 (18.75%) patients had NASH-related liver involvement, characterized by fibrosis (9-11 Kpa) and steatosis above grade 3. This rate is slightly higher than previous reports (8-15%) from other cohorts [4,16,17], possibly reflecting a rising metabolic risk profile in our population. Liver biopsy showed macrovascular steatosis, ballooning degeneration, and minimal fibrosis. Two patients improved with steroids, while four responded to vitamin E treatment.

SLE patients are prone to vascular events, including hepatic vascular complications [8,19,20]. The underlying pathogenesis involves thrombosis, endothelial dysfunction, and hypercoagulability, with clinical presentation varying and imaging studies crucial for diagnosis [8,18]. Our patient with Budd-Chiari syndrome presented with acute severe hepatitis-like illness and ascites. Anticoagulation and supportive treatment led to improvement. Another patient with hepatic infarction, however, succumbed to the condition despite intensive care. A third patient with PVT and gut gangrene improved with anticoagulation and supportive treatment.

One of our patients presented with extrahepatic portal vein obstruction (EHPVO) and massive splenomegaly, a rare association previously reported in SLE patients [19,20]. EHPVO in SLE may be attributed to vasculitis, thrombosis, or other disease-related mechanisms. The patient underwent successful endoscopic variceal ligation and is currently stable on anticoagulation therapy.

DILI remains a significant challenge in the management of SLE, requiring careful balancing of therapeutic efficacy with potential hepatotoxicity. Tailoring treatment regimens, adjusting drug dosages, or discontinuing implicated medications are strategies employed to mitigate DILI while maintaining effective SLE management [3,4]. DILI is a concern in SLE management, with approximately 10%-20% of patients experiencing hepatotoxicity from medications such as corticosteroids, azathioprine, and hydroxychloroquine [3,4]. We had five (15.62%) patients with DILI, which is within the range reported in other studies [3,4], emphasizing the importance of regular monitoring. In all our patients, azathioprine was identified as the causative agent. It was subsequently discontinued and substituted with mycophenolate mofetil, resulting in normalization of liver function tests within three to eight weeks.

SLE-unrelated liver involvement

Consistent with existing literature, two (6.25%) out of 32 patients with SLE-related liver involvement were diagnosed with viral hepatitis in our study [3,4]. Both patients had lupus nephritis and were on renal replacement therapy, highlighting the need for thorough screening for viral hepatitis in SLE patients, particularly those with underlying renal disease or immunosuppression. One patient was HBsAg-positive with CLD and fibrosis and was started on tenofovir alafenamide. The second patient was anti-HCV positive with high viral loads, but no CLD, and improved with a combination treatment with sofosbuvir and velpatasvir.

It is pertinent to say that one patient with ascites was initially suspected to have CLD but was ultimately diagnosed with Tjalma syndrome/pseudo-pseudo Meigs syndrome, a rare clinical entity characterized by narrow-gradient ascites, pleural effusion, and elevated CA 125 levels without ovarian tumor [21,22]. A thorough workup, including peritoneal biopsy, was essential in excluding CLD and establishing the diagnosis. While highlighting positive findings is crucial, it is equally essential to discuss close differential diagnoses that can masquerade as liver involvement, ensuring a comprehensive understanding and accurate diagnosis.

Although not observed in our study, SLE is associated with a diverse spectrum of primary liver diseases, encompassing granulomatous hepatitis, giant cell hepatitis, and chronic hepatitis with immunoglobulin deficiencies [13]. Rare conditions, such as porphyria, idiopathic portal hypertension, and lymphoma, have also been documented. Furthermore, SLE patients are vulnerable to infections such as Listeria monocytogenes [20], Cryptococcus, Candida, and Aspergillus [23], and our classification framework incorporates these conditions to provide a comprehensive overview of SLE-related liver involvement.

Overall, after excluding specific causes, liver dysfunction in SLE patients is primarily driven by disease activity, correlating with markers such as anti-DNA antibodies and SLEDAI (Systemic Lupus Erythematosus Disease Activity Index). Elevated liver enzymes often reflect disease activity, responding to immunosuppression and prednisone. Our study's limitations restrict prevalence elucidation, but our frequency-based findings align with previous data. Variability in SLE liver involvement etiologies may stem from referral bias and limited sample size. We aimed to highlight diverse presentations and propose a subcategorization framework based on literature and clinical experience.

Liver Involvement in SLE: A Comprehensive Classification

SLE-Related Liver Involvement

Liver disease directly attributable to SLE primarily manifests as parenchymal or vascular involvement. Parenchymal disease encompasses lupus hepatitis, AIH overlapping with SLE, and PBC or AIH-PBC overlap syndromes. Vascular involvement may result from direct vessel wall pathology, such as vasculitis or arteritis, or from flow-related abnormalities including hepatic congestion, thrombosis, and vascular proliferation, which can manifest as peliosis hepatis or hepatic hemangioma. Additionally, NRH may arise secondary to portal vasculopathy or as a consequence of long-term immunosuppressive therapy.

Liver Conditions Possibly Associated With SLE

Certain hepatic disorders may occur in the context of SLE without being directly caused by it. NASH may develop due to SLE-related metabolic alterations or coexist with metabolic syndrome. Likewise, SLE may predispose patients to thrombotic complications such as PVT or BCS, though these associations are not firmly established. DILI, while not intrinsically linked to the pathogenesis of SLE, poses a considerable therapeutic challenge, requiring clinicians to balance effective disease control with the risk of liver toxicity.

Liver Conditions Unrelated to SLE

Several hepatic disorders may coexist with SLE but are etiologically independent. These include viral hepatitis (particularly hepatitis B and C) and opportunistic hepatic infections caused by organisms such as *Listeria monocytogenes*, *Cryptococcus*, *Candida*, and *Aspergillus*.

Drawbacks of this study

First, the small sample size limits the generalizability of our findings and precluded the use of inferential statistical analysis or meaningful subgroup comparisons. Moreover, as a retrospective, single-center study, there is a possibility of referral bias, and the absence of detailed longitudinal data on SLE disease activity indices limited our ability to explore correlations between liver involvement and disease flareups or treatment response.

Advantages

A key strength of our methodology is the rigorous patient selection process. We systematically excluded alternative causes through comprehensive serologic testing and targeted liver biopsies, enabling a more accurate subcategorization of liver involvement in SLE patients.

Recommendations

To better understand liver involvement in SLE, larger, homogeneous studies are needed to identify predictors and treatment responses. Collaborative research initiatives can elucidate epidemiology, underlying mechanisms, and develop targeted therapies, ultimately enhancing patient outcomes and care through a multidisciplinary approach.

## Conclusions

Hepatic manifestations in SLE result from a complex interplay of autoimmune mechanisms, vascular complications, and drug-related hepatotoxicity. Recognizing diverse presentations and diagnostic complexities is crucial for clinicians. A collaborative approach between rheumatologists, hepatologists, and specialists is essential for optimizing patient outcomes. Our study highlights the complexity and heterogeneity of liver involvement in SLE, necessitating a comprehensive classification framework. Rigorous diagnostic evaluation is necessary to exclude alternative causes. The study's findings underscore the importance of tailored management strategies for SLE patients with liver involvement, ultimately improving patient care and prognosis.

## References

[1] Petri M, Orbai AM, Alarcón GS (2012). Derivation and validation of the Systemic Lupus International Collaborating Clinics classification criteria for systemic lupus erythematosus. Arthritis Rheum.

[2] Arora S, Isenberg DA, Castrejon I (2020). Measures of adult systemic lupus erythematosus: disease activity and damage. Arthritis Care Res (Hoboken).

[3] Chowdhary VR, Crowson CS, Poterucha JJ, Moder KG (2008). Liver involvement in systemic lupus erythematosus: case review of 40 patients. J Rheumatol.

[4] Huang D, Aghdassi E, Su J (2012). Prevalence and risk factors for liver biochemical abnormalities in Canadian patients with systemic lupus erythematosus. J Rheumatol.

[5] Piga M, Vacca A, Porru G, Garau P, Cauli A, Mathieu A (2011). Two different clinical subsets of lupus hepatitis exist. Mimicking primary autoimmune liver diseases or part of their spectrum?. Lupus.

[6] Shizuma T (2015). Clinical characteristics of concomitant systemic lupus erythematosus and primary biliary cirrhosis: a literature review. J Immunol Res.

[7] Liu Y, Yu J, Oaks Z (2015). Liver injury correlates with biomarkers of autoimmunity and disease activity and represents an organ system involvement in patients with systemic lupus erythematosus. Clin Immunol.

[8] Solela G, Daba M (2023). Budd-Chiari syndrome as an initial presentation of systemic lupus erythematosus associated with antiphospholipid syndrome: a case report with review of the literature. Open Access Rheumatol.

[9] Ferraioli G, Soares Monteiro LB (2019). Ultrasound-based techniques for the diagnosis of liver steatosis. World J Gastroenterol.

[10] Grover S, Rastogi A, Singh J, Rajbongshi A, Bihari C (2014). Spectrum of histomorphologic findings in liver in patients with SLE: a review. Hepat Res Treat.

[11] Arnett FC, Reichlin M (1995). Lupus hepatitis: an under-recognized disease feature associated with autoantibodies to ribosomal P. Am J Med.

[12] Patel S, Demory Beckler M, Kesselman MM (2019). Lupus and the liver: a case study. Cureus.

[13] Perl A (2013). Oxidative stress in the pathology and treatment of systemic lupus erythematosus. Nat Rev Rheumatol.

[14] Kuramochi S, Tashiro Y, Torikata C, Watanabe Y (1982). Systemic lupus erythematosus associated with multiple nodular hyperplasia of the liver. Acta Pathol Jpn.

[15] Vaiphei K, Bhatia A, Sinha SK (2011). Liver pathology in collagen vascular disorders highlighting the vascular changes within portal tracts. Indian J Pathol Microbiol.

[16] Matsumoto T, Yoshimine T, Shimouchi K, Shiotu H, Kuwabara N, Fukuda Y, Hoshi T (1992). The liver in systemic lupus erythematosus: pathologic analysis of 52 cases and review of Japanese Autopsy Registry Data. Hum Pathol.

[17] Ebert EC, Hagspiel KD (2011). Gastrointestinal and hepatic manifestations of systemic lupus erythematosus. J Clin Gastroenterol.

[18] Mukai M, Bohgaki T, Notoya A, Kohno M, Tateno M, Kobayashi S (2000). Liver dysfunction due to apoptosis in a patient with systemic lupus erythematosus. Lupus.

[19] Danowski A, de Azevedo MN, de Souza Papi JA, Petri M (2009). Determinants of risk for venous and arterial thrombosis in primary antiphospholipid syndrome and in antiphospholipid syndrome with systemic lupus erythematosus. J Rheumatol.

[20] Semela D (2015). Systemic disease associated with noncirrhotic portal hypertension. Clin Liver Dis (Hoboken).

[21] Tjalma WA (2005). Ascites, pleural effusion, and CA 125 elevation in an SLE patient, either a Tjalma syndrome or, due to the migrated Filshie clips, a pseudo-Meigs syndrome. Gynecol Oncol.

[22] Torres Jiménez AR, Solís-Vallejo E, Céspedes-Cruz AI, Zeferino Cruz M, Rojas-Curiel EZ, Sánchez-Jara B (2019). Tjalma syndrome (pseudo-pseudo Meigs') as initial manifestation of juvenile-onset systemic lupus erythematosus. Reumatol Clin (Engl Ed).

[23] Kimura M, Udagawa S, Shoji A, Kume H, Iimori M, Satou T, Hashimoto S (1990). Pulmonary aspergillosis due to Aspergillus terreus combined with staphylococcal pneumonia and hepatic candidiasis. Mycopathologia.

